# The Influence of Technology Delivery Mode on Intervention Outcomes: Analysis of a Theory-Based Sexual Health Program

**DOI:** 10.2196/10398

**Published:** 2018-08-29

**Authors:** Nicole Levitz, Erica Wood, Leslie Kantor

**Affiliations:** ^1^ Planned Parenthood Federation of America New York, NY United States; ^2^ Rutgers School of Public Health Rutgers University Newark, NJ United States

**Keywords:** sexual and reproductive health, public health, text messaging, instant messaging, behavior theory, internet

## Abstract

**Background:**

There are few studies on the role of technology delivery mode on health intervention outcomes. Furthermore, the opportunity to examine potential mode effects on a program that is theory-based and integrates principles of communication and decision-making science to influence sexual and reproductive health outcomes is a new contribution to the literature.

**Objective:**

Planned Parenthood Federation of America’s national Chat/Text program can be accessed via short message service (SMS; more commonly referred to as text messaging), Web-based desktop chatting, and mobile phone chatting. The program has been in existence since 2010 and has conducted over 1,000,000 conversations. In this study, we examined whether the mode used to access the program (SMS text, desktop chat, or mobile phone chat) affected program users’ intention to act on the action plan established in their conversation.

**Methods:**

Data were examined for a 6-month period from January 2016 to June 2016. The data were collected as a part of the monitoring and evaluation of an ongoing program. We limited our sample to the program’s priority audience of 15-24 years residing within the United States, which resulted in a sample of 64,939 conversations. Available data items for analysis included user demographics, delivery mode, topic discussed, helpfulness rating (on a 4-point scale), user confidence in following through on the intentions made during the conversation (on a 4-point scale), and educator confidence in whether the user would follow through on the stated intention. Linear and multinomial robust regression analyses were conducted to examine the relationships between conversation delivery mode and confidence.

**Results:**

No significant relationships between users’ confidence to carry out their intentions and gender or race were found. None of the 3 modalities (SMS text, desktop chat, or mobile phone chat) were significantly associated with user confidence. All the 3 modalities had significant associations with educator confidence and showed similar effect sizes to those of user confidence. Educator confidence was significantly associated with all the topics discussed.

**Conclusions:**

The Planned Parenthood Chat/Text program was designed as a tool to improve access to sexual and reproductive health care among young people. The mode of intervention delivery was not associated with users’ confidence in their ability to carry out their stated intention, suggesting that all modes are legitimate for delivering this intervention. Furthermore, each mode worked across gender and race or ethnicity, indicating that this is a modality that can work across groups.

## Introduction

The nearly ubiquitous use of the internet among adolescents and young adults in the United States has created a potential platform for health programs to reach these populations. Over 75% of teenagers have a smartphone, and an estimated 93% of teenagers in the United States are users of the internet [1]. Many of these adolescents and young adults use Web and mobile phone technology to find health-related information. Northwestern University found that 84% of teenagers searched health information on the Web [2] and that 34% of these individuals reported a change in health behavior because of Web-based health information [3]. However, many digitally-based health programs to date lack a theoretical structure despite evidence that greater effect sizes are observed on intended populations from interventions with a theoretical underpinning as compared with those without one [3-6]. Including behavioral theory within program design helps to address the complex psychological processes that underlie the execution of behavior and is important for the creation of health programs that contribute to sustainable behavioral change. One theory, in particular, that has been underutilized in Web- and mobile phone-based program design is the unified theory of behavior (UTB) [7,8].

The UTB provides a valuable framework for conceptualizing the complex pathway that underlies behavior. This theory emphasizes the determinative role that intention plays in the execution of behavior and draws on several interconnected constructs that affect the formation of intention and that mediate the relationship between intention and behavior [9]. The key constructs that influence the formation of intention are (1) the perceived advantages and disadvantages of performing the behavior, (2) social norms surrounding the behavior, (3) social image repercussions (ie, does this behavior align with the view people hold of themselves?), (4) emotions and affect toward the behavior, and (5) self-efficacy. However, a set intention does not always translate into behavior. Rather, it is mediated by the (1) knowledge and skills needed to carry out the behavior, (2) environmental constraints or facilitators, (3) salience of the behavior, and (4) automatic and habitual psychological processes [7-9]. Altogether, these variables interact to facilitate or hinder one’s ability to transform intention into behavior.

Planned Parenthood Federation of America’s national Chat/Text Program (the program) incorporates behavioral science by honing in on several of the key UTB constructs that contribute to the formation of intention and by using communication theory to structure conversations to encourage healthy sexual and reproductive health behaviors. Elements of the communication theory included in the program focus on establishing trustworthiness, expertise, and accessibility by the health educators; these elements have a long history in the communication literature and have been found to influence attitude change [10]. Trustworthiness is established through a name exchange; a statement showing expertise is shared immediately following the name exchange, and accessibility is demonstrated by sharing local health center information and proactively inviting users to come back whenever they want.

The program is staffed by trained health educators who freely tailor scripted message library health information to individual user’s needs. The scripted message library is a tool for educators to rely on to ensure the accuracy and consistency of health information shared. Educators are trained on health content and how to apply communication and behavioral science, initially with a robust, in-person 40-hour training and then quarterly booster trainings thereafter or more frequently if the quality assurance process identifies a gap or need.

Program conversations are structured to begin with the health educator establishing trustworthiness, expertise, and accessibility with the user and then working with the user to explicitly identify their health concerns and goals. These concerns and goals are then incorporated into the construction of a personalized action plan to address their specific sexual and/or reproductive health concerns. Within each conversation, health educators typically address several UTB constructs, including (1) beliefs surrounding the health behavior in discussion, (2) emotions related to this behavior, (3) self-image, and (4) self-efficacy. In addition, the program incorporates measures that capture users’ intentions to act on action plans discussed in conversations. At the end, health educators have the option to ask users to state their intended action plan for addressing their specific health concerns and how confident they are in executing this plan. Once the intention is affirmed by the user, the program helps the user to make a plan that will help ensure that intentions are translated into behavior by working with the user through habits and environmental constraints while addressing knowledge and obstacles. Obstacles that are frequently reported by users are transportation or cost, and health educators can typically assist users with addressing these concerns. Knowledge and skills are generally addressed with how to execute the intention, such as how to take birth control pills at the same time each day. Using the UTB as a framework, confidence in the intention to act on the personalized plan is used as a proxy for behavior because intention is theorized to be its strongest predictor [7-9].

Each month, the program conducts approximately 20,000 conversations with users, most of whom find the program using search and come through the organization’s website. The average user identifies as an 18-year-old female, and about half of the users identify as people of color. Each conversation takes an average of 15 min (mean, median, and mode), and there is no time constraint placed on the conversations. Quality assurance is conducted on an on-going basis to assure that educators follow the behavioral science-informed protocol consistently. A team of 5 experienced educators score 3% of all conversations each month on a 19-point metric that includes all key elements of the protocol.

There has been little focus on the impact of the mode of a digitally-based health intervention (eg, text messaging vs Web-based design) on health programs’ intended outcomes. Because the program can be accessed via text messaging or short message service (SMS), Web-based desktop chatting, or mobile phone chatting, we sought to examine how a user’s choice of mode for accessing the program affected their intention to act on the action plan established in their conversation. In this study, we examined the effects of mode of delivery on both the user’s and the health educator’s confidence in executing the user-determined intention set during the course of the conversation. Asking more probing questions to hit on all 5 intention factors may lead to improved outcomes. Adherence to theory within the intervention via content analysis and reviewing differences in effects may clarify how theory can translate into the digital sphere and how it can translate back into outcomes. In addition to examining intentions, we looked at the effects that confidence had on moderating the relationship between mode of delivery and perceived helpfulness by the user.

## Methods

### Data Collection

Data were collected over a 6-month period from January 2016 to June 2016 as part of the monitoring and evaluation of the ongoing program. Three surveys were the main sources of data for this study. Pre- and postconversation surveys were filled out by the users and a postsurvey was filled out by the health educators. The presurveys gathered users’ demographic information and were required for all Web-based users but were optional for text users. The user postsurvey is optional and asks the user to evaluate how helpful the program was. The educator postsurveys included measures for educators to capture the level of confidence the user expressed in executing the discussed action plan when users were asked about it explicitly during the course of the conversation. If users did not share a response about confidence, their data were excluded. Educators also had to indicate how confident they were in the user’s ability to act on their plan. In addition, health educators were required to indicate the sexual and reproductive health topics discussed throughout the course of each chat in the postsurvey. As the data were deidentified and did not contain personal health information, this study did not undergo review by an institutional review board.

### Measures

Demographic information was obtained from the user prechat surveys. However, demographic data collection varied according to the mode of delivery. Desktop and mobile phone users were required to complete these prechat fields before speaking to a health educator, whereas SMS (text) users were asked for this information but it was not compulsory to provide it before using the service.

Educators recorded the topics discussed during the conversation in their postsurveys. Topics included birth control, emergency contraception, sexually transmitted infection (STI) testing, pregnancy testing, abortion, and other. All topics except for *other* were categorized as dichotomous (*yes discussed* and *no not discussed*). The *other* selection was collected via a qualitative open field, but the specific entries were not analyzed in this study.

Confidence in the intention to follow through with personalized action plans was measured during and after the conversation by both the user and the educator, respectively. A 4-point Likert scale ranging from *not at all confident* to *very confident* was sent to users by the health educator before the conclusion of the conversation. Users directly indicated to the educator their level of confidence, and the educator then reported their response in their postsurvey. This measure was not used in the final model because of missing data. The educator’s confidence scale that measured perceived confidence in the user’s intention of acting on the plan discussed was the same 4-point Likert scale. These confidence scales were reverse coded so that higher scores reflected greater confidence (ie, *not at all confident* had a value of 0 and *very confident* had a value of 3). There were also options included for the health educators to indicate if *no next steps [were] discussed* and if the message was not sent but were not excluded from these analyses. The measures of intention were designed for this study.

### Analysis

The sample was limited to the program’s target audience of 15- to 24-year-olds residing in the United States and excluded users identifying as genders other than male or female, as they made up less than 0.50% (328/65,627) of users. The sample consisted of 64,939 conversations between January and June 2016.

Descriptive statistics, bivariate analyses, and linear regression models were all conducted using IBM SPSS (SPSS Inc, Chicago, IL) Version 21. Correlations were run between demographic variables, topic discussed, user confidence, educator confidence, mode of delivery, and helpfulness. The linear models regressed topics discussed and delivery modes used onto confidence and were created for both user confidence in the execution of their behavioral intention set during the course of conversation and educator confidence in the user executing this behavioral intention. Linear models were adjusted for user age, gender, and race or ethnicity.

**Figure 1:**
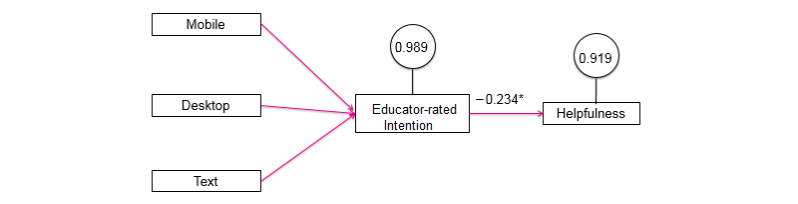
Mediation model examining the path from modality to helpfulness. Note that 0.989 is the disturbance term for educator-rated intention, and 0.919 is the disturbance term for helpfulness.

MPlus (Muthén & Muthén, Los Angeles, CA) Version 7.11 was used to conduct the multinomial robust regression analyses. We wanted to see if the topic of conversation moderated the level of confidence an individual user felt by delivery mode (see Figure 1 for path model). This modeling also provided the ability to connect helpfulness and examine its interplay with confidence as a consecutive sequence. First, the user chooses a mode of delivery, and then, they have a conversation about a given topic, establish an action plan, and identify a certain level of confidence in that plan and the degree of helpfulness overall.

## Results

### Sample

Table 1 displays details about the demographic composition of the sample. Demographic data are sorted by modality to further explicate the factors that contribute to setting a behavioral intention in the course of a conversation. The 3 modes of delivery to use the program are SMS text message, desktop Web-based chat, and mobile phone Web-based chat.

### Bivariate Analysis

Correlations were run between demographic variables, topic discussed, user confidence, educator confidence, mode of delivery, and helpfulness. Most of the observed relationships were close to zero and only a few were significant. Significant relationships were observed between user confidence and educator confidence (*r*=.82, *P*<.001) and between user confidence and helpfulness (*r*=.39, *P*<.001). Users who identified as female constituted the vast majority of the user base during this period; however, there were no significant gender differences by mode of delivery. Users who identified as Hispanic or Latino were 1.8 times more likely to access the program via mobile phone messaging as compared with users who identified as white. Similarly, users who identified as black were 1.6 times more likely to access the program via mobile phone than whites. Users who identified as Asian were 1.8 times more likely to be desktop users as compared with whites. Users who identified as American Indian or Alaska Native or as *other* race or ethnicity category were over 5 times more likely to be text users as compared with users who identified as whites. No other significant demographic distinctions by mode of delivery were observed.

Observed correlations between age groups and topics discussed were close to zero and nonsignificant. There were no significant differences in the observed relationships for each of the 5 main sexual health topics the health educators discuss (ie, birth control, pregnancy testing, abortion, STI testing, and emergency contraception) by age, gender, and race or ethnicity and user confidence, educator confidence, mode of delivery, and helpfulness. These correlations were also all close to zero.

### Linear Regressions

No significant relationships between user confidence and gender or race were found (see Table 2 below). However, there were statistically significant differences in educator confidence by both user race and gender. Educators had minimally higher confidence in users who identified as Hispanic (beta=.043, *P*=.002) and multiracial (beta=.065, *P*=.003) as compared with whites.

Age did have a small but significant effect on both user and educator confidence. The older the user, the higher the confidence in executing the intention from both the user’s (beta=.28, *P*<.001) and educator’s perspective (beta=.27, *P*<.001).

**Table 1:** Background data for respondents by modality.

Characteristics		Number of users by modality, n (%)		
		Mobile phone Web-based chat (n=34,136)	Desktop Web-based chat (n=30,328)	Short message service text message (n=475)
**Gender**				
	Female	30,841 (90.35)	27,248 (89.84)	423 (89.05)
	Male	3295 (9.65)	3080 (10.16)	52 (10.95)
**Race or ethnicity**				
	White	15,621 (47.37)	16,419 (54.55)	208 (46.43)
	African American or black	4670 (14.16)	3192 (10.61)	50 (11.16)
	Hispanic or Latino	8291 (25.14)	4851 (16.12)	67 (14.96)
	Asian	1546 (4.69)	3046 (10.12)	55 (12.28)
	Hawaiian Islander	188 (0.57)	204 (0.68)	0 (0.0)
	American Indian or Alaska Native	174 (0.53)	147 (0.49)	11 (2.46)
	Multiracial	2487 (7.54)	1868 (6.21)	44 (9.82)
	Other	0 (0.0)	370 (1.23)	448 (2.90)
**Age group (years)**				
	15 to 19	22,479 (65.85)	17,774 (58.61)	352 (74.11)
	20 to 24	11,657 (34.15)	12,554 (41.39)	123 (25.89)

**Table 2:** Multivariate regression models predicting user and educator confidence.

Mode of intervention^a^		Beta	Standard error	Margins of error	*P* value
**Mobile phone chat (compared with text)**					
	User	−.210	0.130	±0.25	.11 (NS^b^)
	Educator	−.226	0.065	±0.13	.02
**Text (compared with desktop)**					
	User	.255	0.130	±0.25	.05 (NS)
	Educator	.159	0.065	±0.13	.02
**Desktop chat (compared with mobile phone)**					
	User	.045	0.028	±0.05	.10 (NS)
	Educator	.067	0.011	±0.22	<.001
**Abortion**					
	User	.070	0.042	±0.08	.10 (NS)
	Educator	.212	0.018	±0.04	<.001
**Birth control**					
	User	.171	0.031	±0.06	<.001
	Educator	.171	0.012	±0.02	<.001
**Emergency contraception**					
	User	−.016	0.032	±0.06	.67 (NS)
	Educator	.036	0.014	±0.03	.01
**Pregnancy testing**					
	User	−.026	0.027	±0.05	.34 (NS)
	Educator	.058	0.012	±0.03	<.001
**STI^c^ testing**					
	User	.070	0.029	±0.06	.02
	Educator	.171	0.014	±0.03	<.001
**Other**					
	User	−.024	0.054	±0.11	.66 (NS)
	Educator	−.103	0.021	±0.04	<.001	

^a^Models above control for age, race or ethnicity, and gender.

^b^NS: not significant.

^c^STI: sexually transmitted infection.

About half of the users selected to access the program via mobile phone chat and the other half by desktop. Only 0.70% (475/64,939) of users accessed the program via SMS. All 3 modes were not significantly associated with user confidence. Desktop chat had a nonsignificant negative effect on confidence compared with mobile phone (beta=−.210, *P*=.11), and mobile phone chat was marginally significant with a positive effect (beta=−.255, *P*=.05). All 3 modes of delivery had significant associations with educator confidence and showed similar effect sizes as user confidence. All effects were very small changes on the 4-point Likert scale.

Across all 3 modes, helpfulness gives a sense of overall program satisfaction, 52.44% (4914/9371) strongly agreed and 31.35% (2938/9371) agreed the conversation was helpful. In addition, 7.50% (703/9371) disagreed it was helpful and 8.70% (815/9371) strongly disagreed it was helpful. User confidence in executing the action plan they came up with as part of the conversation was generally high. Overall, 63.99% (1964/3069) of users were very confident, 23.17% (711/3069) were somewhat confident, 12.09% (371/3069) were a little confident, and 0.75% (23/3069) were not at all confident. Educators’ confidence in user execution of their action plan was lower than that of the users’ own evaluation of confidence. Among educators, 18.02% (3943/21,877) were very confident, 45.7% (10,005/21,877) were somewhat confident, 30.51% (6674/21,877) were a little confident, and 5.74% (1255/21,877) were not at all confident. Only 33.69% (21,877/64,939) of conversations got to the end of the protocol so that educators could evaluate confidence.

Birth control and STI testing were the only topics that significantly affected user confidence. Educator confidence was significantly associated with all of the topics discussed. However, none of these associations were very strong. Users were less confident about next steps after emergency contraception and pregnancy testing conversations, but this relationship was not observed among the educators’ responses. Both users and educators had reduced confidence in conversations that discussed topics outside the program scope (ie, *other* topics).

### Mediation Model

To take these analyses one step further, we explored a model that regressed mode of delivery onto confidence and confidence onto helpfulness. The addition of helpfulness was to help further assess intention as a proxy of behavior to see whether the theory-based protocol supported users in achieving their immediate goals with the program. As we found with the regressions, there was a great deal of missing data on user intention. We first tried the model using user intention, but there were insufficient data to complete the covariance matrix, and hence, we moved to relying on the educator-reported intention. The covariance matrix needs to be at 0.5 or above to invoke full information maximum likelihood, and user confidence was below this threshold at 0.05. We found the user and educator intention measures to be correlated 0.95 and as such felt comfortable with the use of this proxy. Unfortunately, there was a similar problem with the helpfulness measure and missing data, but there was no logical proxy for helpfulness, so we ran the model with it (see Figure 1 for the final model).

We found a just identified model in which the number of free parameters exactly equals the number of known values (ie, a model with 0 degrees of freedom). As such, no assessment of model fit is possible. In addition, the joint significance test failed for mobile phone and desktop, so we could not move forward with the interpretation of these paths. Text use did pass the joint significance test. The total effect size was 0.857, but these results must be interpreted with a high level of caution given the missing data.

## Discussion

### Principal Findings

The vast use of the internet and mobile devices has allowed for the creation of health interventions and programs designed to increase health-promoting behaviors among the most vulnerable populations in need of health care access, such as youth of color living in economically disadvantaged areas [3,11]. This program was designed as a tool to improve access to sexual and reproductive health care among young people, and we were eager to see if it could be used as a tool to promote health equity. The equality effects indistinguishable by identity seen among the observed relationships between confidence and across racial and ethnic groups indicate that it can be used as a tool to promote health equity. Previous research conducted by the Pew Research Center found differences in the use of internet and mobile technology by race and ethnicity [1]. African-Americans and Latinos are more likely to rely on the internet for health information than whites [1]. Furthermore, African-Americans and Latinos are more likely to rely on their mobile phones for internet access as compared with whites [1]. These differences are further supported by a process evaluation of this program conducted by Giorgio et al, who found that there are significant demographic differences between those using mobile phone chat and SMS versus desktop chat to access the program [12]. Although we did not see significant differences here in the demographics accessing the program by mode, it is important to consistently monitor and assure that this program is equally accessible to help assure greater access to the populations that need it most.

The lack of effects from these analyses tells an important story. Most of the observed relationships between mode of delivery and intention were close to zero. The strongest observed relationships were between user and educator confidence in following through on intentions and how helpful the user perceived the program to be. This association indicates that intention setting can help meet users’ needs. Small, negative correlations were observed among user confidence and the following 3 topics: birth control, STI testing, and pregnancy testing. However, these correlations fall out with more rigorous analysis. In addition, race and gender do not appear to play a significant role in how intention setting works for users.

In general, the older the user, the higher their confidence in executing intention. However, the program leads to essentially the same confidence in intention to follow through on their action plan no matter the user’s gender or race or ethnicity. This equality observed by race or ethnicity and gender in user confidence and the minimal effects of these variables on educator confidence indicate that program protocol resonates with users and helps them set an intention they believe they can execute across gender and race or ethnicity. The program was designed for young people aged 15 to 24 years of all identities.

Mode of delivery led to minimal changes on the 4-point Likert scale, indicating that mode does not significantly affect user confidence. Although there were small associations with Hispanic users using text more and Asian users using desktop chat more, mode did not appear to play a role in how the intervention was received. No matter which mode a user came through, they were just as likely to set an action plan that they intended to execute. This finding adds to a limited literature and is important in a world where there is increasing interest in developing digital interventions. This investigation suggests that program developers need to focus on the underlying theoretical approach of interventions and not simply on the mode of delivery when creating interventions.

### Limitations

Use of existing program data comes with limitations. We would have liked to examine confidence with more nuance and consistency, but the measures are preset. Although the 4-point metric is considered sufficient for quality assurance, the models here may require scales with more points to illuminate other differences. Furthermore, users often exit the program before making their way through the entire program protocol. Only approximately 60% of users make it all the way through. Thus, many users have not had the opportunity to set an intention, build an action plan, and discuss obstacles and are therefore never asked about their confidence after the conversation has ended. In addition, as the educator sees the user’s response to the confidence question before entering their own, it is inherently biasing their response.

In addition, there were several technological limitations that affected the data collection process. The SMS platform used did not require that SMS users complete survey questions before or after entering a conversation, leaving us with even more missing data for this mode than the other modes. The postchat survey for desktop and mobile phone users was also not mandatory, and users had to opt in for completion, leaving us with another significant source of missing data. We were also limited to the existing cross-sectional data and could not follow-up with users to check if they executed their intended health behaviors. More robust analyses are required using a more complete dataset to further examine the relationships between these variables.

Generalizability of these findings is limited to only the program itself. Mode of delivery and topic may play a different role in other digital health programs using different protocols, theory-based or not.

## Abbreviations

SMS: short message service
STI: sexually transmitted infection
UTB: unified theory of behavior
